# Common Variation in Vitamin D Pathway Genes Predicts Circulating 25-Hydroxyvitamin D Levels among African Americans

**DOI:** 10.1371/journal.pone.0028623

**Published:** 2011-12-21

**Authors:** Lisa B. Signorello, Jiajun Shi, Qiuyin Cai, Wei Zheng, Scott M. Williams, Jirong Long, Sarah S. Cohen, Guoliang Li, Bruce W. Hollis, Jeffrey R. Smith, William J. Blot

**Affiliations:** 1 International Epidemiology Institute, Rockville, Maryland, United States of America; 2 Division of Epidemiology, Department of Medicine, Vanderbilt Epidemiology Center, Vanderbilt University School of Medicine and the Vanderbilt-Ingram Cancer Center, Nashville, Tennessee, United States of America; 3 Division of Human Genomics, Department of Molecular Physiology and Biophysics, Vanderbilt University, Nashville, Tennessee, United States of America; 4 Department of Pediatrics, Medical University of South Carolina, Charleston, South Carolina, United States of America; 5 Division of Genetic Medicine, Department of Medicine, Vanderbilt University Medical Center, Nashville, Tennessee, United States of America; University of California, Berkeley, United States of America

## Abstract

Vitamin D is implicated in a wide range of health outcomes, and although environmental predictors of vitamin D levels are known, the genetic drivers of vitamin D status remain to be clarified. African Americans are a group at particularly high risk for vitamin D insufficiency but to date have been virtually absent from studies of genetic predictors of circulating vitamin D levels. Within the Southern Community Cohort Study, we investigated the association between 94 single nucleotide polymorphisms (SNPs) in five vitamin D pathway genes (*GC*, *VDR*, *CYP2R1*, *CYP24A1*, *CYP27B1*) and serum 25-hydroxyvitamin D (25(OH)D) levels among 379 African American and 379 Caucasian participants. We found statistically significant associations with three SNPs (rs2298849 and rs2282679 in the *GC* gene, and rs10877012 in the *CYP27B1* gene), although only for African Americans. A genotype score, representing the number of risk alleles across the three SNPs, alone accounted for 4.6% of the variation in serum vitamin D among African Americans. A genotype score of 5 (vs. 1) was also associated with a 7.1 ng/mL reduction in serum 25(OH)D levels and a six-fold risk of vitamin D insufficiency (<20 ng/mL) (odds ratio 6.0, p = 0.01) among African Americans. With African ancestry determined from a panel of 276 ancestry informative SNPs, we found that high risk genotypes did not cluster among those with higher African ancestry. This study is one of the first to investigate common genetic variation in relation to vitamin D levels in African Americans, and the first to evaluate how vitamin D-associated genotypes vary in relation to African ancestry. These results suggest that further evaluation of genetic contributors to vitamin D status among African Americans may help provide insights regarding racial health disparities or enable the identification of subgroups especially in need of vitamin D-related interventions.

## Introduction

External exposures (sunlight, diet, and vitamin supplements) are chief determinants of circulating 25-hydroxyvitamin D (25(OH)D), the established clinical marker of vitamin D status [1]. However, with several genes controlling pathways that synthesize, transport, and degrade forms of vitamin D, common genetic variants may also play a role in individual (and potentially population) differences in vitamin D status. It is increasingly appreciated that vitamin D status is to some extent under genetic control [2], and that the heritability of vitamin D levels may be greater than previously assumed [3]–[7]. With vitamin D implicated in a wide range of health issues from rickets to cancer [8], [9], a fuller understanding of the determinants of vitamin D status is needed and must include consideration of inherited characteristics.

The group-specific complement gene (*GC*) on chromosome 4 encodes the vitamin D binding protein (VDBP) that is the main transporter of vitamin D and its metabolites in the circulation [10]. Evidence is accumulating that polymorphisms in the *GC* gene are associated with circulating 25(OH)D levels [2]. Recently, two genome-wide association studies (GWAS) of 25(OH)D levels in populations of European ancestry found strong evidence for an association with a single nucleotide polymorphism (SNP) in *GC* (rs2282679 in intron 12) [11], [12]. The *GC* SNPs rs7041 and rs4588 in exon 11 have also been the subject of much attention in the literature [7], [13]–[19]. Studies are now evaluating the independent or 25(OH)D-effect-modifying association between *GC* SNPs and disease outcomes [14], [17]–[19], [20]–[23]. In addition, there are several other vitamin D pathway genes (e.g., *CYP27B1*, *CYP24A1*, *CYP2R1*, *VDR*) that may contribute to the genetic basis of vitamin D status, but which have been less studied.

African Americans have considerably lower average 25(OH)D levels than Caucasians in the United States, with a prevalence of vitamin D insufficiency reported in the range of 40–80% [24]–[29]. We have previously reported on racial differences in vitamin D status [24] and recently found novel associations between the degree of African ancestry and serum 25(OH)D levels among African Americans in the Southern Community Cohort Study (SCCS), for which explanations beyond possible skin color gradations should be explored [30]. Although there is racial variation in the *GC* gene [10], [31], to date the association between *GC* gene polymorphisms and 25(OH)D has been studied, with few exceptions [7], [32], almost exclusively in populations of European descent [11]–[13], [16], [20], [33]. We therefore undertook a study to investigate (1) whether and to what extent genetic polymorphisms in *GC, CYP27B1*, *CYP24A1*, *CYP2R1*, and *VDR* determine 25(OH)D levels in African Americans and Caucasians; and (2) whether polymorphisms in these genes mediate the association between African ancestry and 25(OH)D levels among African Americans that we previously reported [30].

## Results

Characteristics of the study population are presented in Table 1. African Americans had lower mean serum 25(OH)D levels (17.5 ng/mL, 95%CI 16.6–18.4 ng/mL) than Caucasians (27.2 ng/mL, 95%CI 26.1–28.3 ng/mL). The enrollment of African Americans and Caucasians from the same community health centers year-round resulted in only minor differences in residential ultraviolet radiation (UVR) scores and season of enrollment by race.

**Table 1:** Serum 25(OH)D levels and other related characteristics for 758 African American and Caucasian SCCS participants.

Characteristic	African American (N = 379)	Caucasian (N = 379)
Number of females/males	187/192	187/192
Mean (range) percentage African ancestry	0.929 (0.505,0.999)	0.009 (0.001,0.171)
Mean (range) percentage European ancestry	0.071 (0.001,0.495)	0.991 (0.829,0.999)
Mean (S.D.) serum 25(OH)D levels, ng/mL	17.5 (8.6)	27.2 (11.1)
Mean (S.D.) age, years	51.9 (9.0)	54.1 (9.5)
Mean (S.D.) body mass index^a^, kg/m^2^	28.3 (5.6)	28.4 (5.8)
Mean (S.D.) daily dietary intake of vitamin D^b^, IU	218 (213)	269 (236)
Mean (S.D.) residential UVR score	5.2 (1.9)	5.2 (2.0)
% enrolled in winter^c^	17%	22%
% enrolled in spring^c^	30%	27%
% enrolled in summer^c^	25%	26%
% enrolled in fall^c^	28%	25%
% current smoker^a^	34%	34%

Abbreviations: SCCS, Southern Community Cohort Study; S.D., standard deviation; IU, International Units; UVR, ultraviolet radiation.

a Selection/matching factor.

b From food sources and multivitamin and calcium supplement sources.

c Winter: December-February; Spring: March-May; Summer: June-August; Fall: September-November.

The SNPs rs222054 in the *GC* gene and rs12314197 in the *VDR* gene deviated from HWE for Caucasians, and these data were dropped from the study. The remaining SNP genotype frequencies and mean serum 25(OH)D levels by genotype among African American and Caucasian subjects are presented in Table S1, with the significant results shown in Table 2. Three SNPs were significantly associated with 25(OH)D, but only among African Americans. Two of the SNPs, rs2298849 and rs2282679, were in the *GC* gene but not in LD with each other (r^2^ = 0.01 in YRI and 0.08 in CEU) and one was in the *CYP27B1* gene (rs10877012). The reference allele frequencies for these SNPs in our African American and Caucasian study groups were very similar to those reported in HapMap for the YRI and CEU populations, respectively (not shown).

**Table 2:** Single nucleotide polymorphisms (SNPs) significantly associated with serum 25(OH)D levels.

Gene	dbSNP ID	Chr	Position^a^	African Americans (N = 379)					Caucasians (N = 379)				
				Homozygous referent (HR), Heterozygous (HET), Homozygous variant (HV)^a^ (*N in parentheses*)	Mean serum 25(OH)D, ng/mL			p-value^b^	Homozygous referent (HR), Heterozygous (HET), Homozygous variant (HV)^a^ (*N in parentheses*)	Mean serum 25(OH)D, ng/mL			p-value^b^
					HR	HET	HV			HR	HET	HV	
***GC***	rs2298849	4	72867715	AA (139), AG (172), GG (61)	16.4	17.5	20.4	**0.008**	AA (240), AG (120), GG (10)	27.1	27.0	27.3	0.97
	rs2282679	4	72827247	TT (317), GT (57), GG (1)	17.7	15.5	9.4	**0.03**	TT (199), GT (150), GG (26)	27.5	27.0	26.9	0.66
***CYP27B1***	rs10877012	12	56448352	GG (291), GT (82), TT (3)	16.9	18.9	26.5	**0.02**	GG (188), GT (161), TT (27)	27.3	27.0	28.0	0.99

a Base position on chromosome (Chr) and allele naming of each single nucleotide polymorphism (SNP) are based on forward strand according to human genome assembly 18 (March 2006, NCBI Build 36.1).

b p-value for the association between the SNP genotypes (parameterized as 0/1/2, for HR/HET/HV, respectively) and 25(OH)D levels from race-stratified linear regression models adjusted for sex and level of African ancestry.

The three identified SNPs were then modeled to estimate the effect of each high risk genotype (Table 3). These analyses showed that variation in each SNP was associated with average 25(OH)D changes of 2.1 to 3.6 ng/mL. When the three SNPs were included in the same model, only rs2298849 retained statistical significance, with the AA genotype associated with an average decrease in serum 25(OH)D of 3.3 ng/mL compared to the GG genotype.

**Table 3:** Linear regression-derived associations between rs2298849, rs2282679, and rs10877012 and serum levels of 25(OH)D among African Americans.

SNP	Comparison	beta^a^	p-value
**Model type 1** b			
rs2298849	AA to GG	−3.62	0.005
rs2282679	(GT, GG combined^c^) to TT	−2.53	0.03
rs10877012	GG to (GT, TT combined^c^)	−2.11	0.04
**Model type 2** d			
rs2298849	AA to GG	−3.27	0.01
rs2282679	(GT, GG combined^c^) to TT	−1.87	0.13
rs10877012	GG to (GT, TT combined^c^)	−1.40	0.18

a Interpreted as the average change in serum 25(OH)D level (ng/mL) associated with the genotype comparison shown in the table.

b Three separate linear regression models including each SNP alone, adjusted for sex and percentage African ancestry.

c Genotypes combined because less than five subjects had the homozygous variant genotype.

d One linear regression model including the three SNPs together, adjusted for sex and percentage African ancestry.

**Figure 1:**
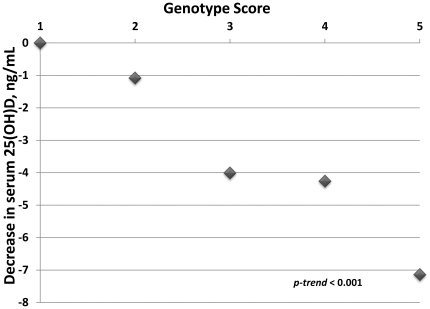
Genotype score in relation to serum 25(OH)D levels among African Americans. Plot of the estimated, average decrease in serum level of 25(OH)D (y-axis) in relation to genotype score (x-axis). Genotype score equals the sum of the number of risk alleles (i.e., A for rs2298849, G for rs2282679, and G for rs10877012). Estimates were derived from a linear regression model restricted to African Americans, using a genotype score of 1 as a referent, and including covariates for sex and level of African ancestry.

A genotype score was constructed as the sum of the number of G alleles for rs2282679, A alleles for rs2298849, and G alleles for rs10877012 (i.e., a sum of the risk alleles). The range of the genotype score was 1–5 for African Americans and 1–6 for Caucasians. This score was strongly and significantly related to 25(OH)D levels among African Americans (Figure 1) but not among Caucasians (not shown). African Americans with a score of 5 had mean serum vitamin D levels 7.1 ng/mL lower than those with a score of 1, and the trend across the score was highly significant (*p_trend_*<0.001). In addition, those with scores 2, 3, 4, and 5 (compared to 1) were at progressively increased risk for vitamin D insufficiency: OR = 1.35 (p = 0.56), 2.67 (p = 0.05), 2.54 (p = 0.06), and 6.01 (p = 0.01), respectively.

Among African Americans, we examined how high risk genotypes for the three SNPs were distributed across tertiles of African ancestry. Overall, we observed that the high risk genotypes for rs2298849, rs2282679, and rs110877012 did not cluster among those of higher African ancestry and so would not seem to predict (on their own) an association between higher African ancestry and lower vitamin D levels (Table 4). Those in the highest tertile of African ancestry were even significantly less likely (p = 0.003) to have the high risk genotype for rs2282679. The average genotype score also varied little across tertiles of African ancestry (3.1–3.2), and a linear regression model estimated that for each 10% increase in African ancestry, genotype score remained virtually unchanged (β = +0.008, p = 0.89).

**Table 4:** Association between level of African Ancestry and genotypes of rs2298849, rs2282679, and rs10877012 among African Americans.

	Frequency of high risk genotype^a^ (%)			
	Lowest tertile African ancestry	Middle tertile African ancestry	Highest tertile African ancestry	p-value^b^
**High risk genotype** a				
AA (rs2298849)	38%	39%	35%	0.83
GG or GT^c^ (rs2282679)	21%	19%	7%	0.003
GG (rs10877012)	74%	79%	79%	0.50
**Mean (S.D.) genotype score**	3.1 (1.1)	3.2 (1.0)	3.1 (0.9)	

a Genotype associated with the lowest 25(OH)D levels in our data.

b p-value from Pearson's chi square test comparing proportions across the three ancestry groups.

c Genotypes combined because less than five subjects had the homozygous variant genotype.

A linear regression model restricted to African Americans, with covariates for dietary vitamin D intake, UVR score, age, body mass index, smoking, sex, and percentage African ancestry accounted for 26% of the variation in 25(OH)D levels (model R^2^ = 0.259). The addition of the genotype score increased the model R^2^ to 0.285, indicating these three SNPs accounted for a 10.0% relative increase in the model's ability to explain variation in 25(OH)D. A likelihood ratio test comparing the two models was also statistically significant (p = 0.02), indicating that the genotype score significantly increased the model's explanatory power for African Americans. Among Caucasians, the addition of the genotype score to a model including the same environmental determinants also increased the model R^2^ from 0.176 to 0.193, although the likelihood ratio test comparing the two models was not statistically significant (p = 0.22).

## Discussion

Although the heritability of vitamin D status appears considerable [3]–[7], the specific genetic determinants of vitamin D levels are only beginning to be identified. Herein, in one of the first studies of genetic determinants of 25(OH)D levels involving African Americans, we found significant associations with three SNPs in vitamin D pathway genes, all of which replicate earlier findings in populations of European ancestry (rs2282679 [11], [12], [20], rs2298849 [33], rs10877012 [34], [35]). Importantly, included among these was rs2282679, a highly significant result from two recent GWAS [11], [12], one of which reported a 49% increased risk for vitamin D insufficiency (<20 ng/mL) associated with the rs2282679 minor allele among Caucasians [12]. Among African Americans, we found the risk of vitamin D insufficiency associated with the GT or GG genotype for rs2282679 to be similarly elevated (OR = 1.70) although not statistically significant (p = 0.12). It is notable that we found no significant associations for Caucasians in our study, the size of which was relatively small, and power limitations might explain why we failed to detect a signal among Caucasians. Because African Americans have lower baseline levels of 25(OH)D than Caucasians and may not benefit equally from sunlight-mediated vitamin D synthesis due to heavy skin melanization, genetic influences on 25(OH)D may be relatively more important in determining overall vitamin D status among African Americans and easier to detect in smaller studies. The only previous study of genetic predictors of 25(OH)D among African Americans involved 513 African Americans from 42 families in Los Angeles, CA, and evaluated 30 SNPs in *GC*, *VDR*, and *CYP27B1* [7]. Although the examined SNPs included two of the three for which we found an association (rs2298849 and rs10877012), significant findings were reported only for the *GC* SNPs rs7041 and rs4588 [7].

Factors related to sunlight (e.g., UVR, season, time spent outdoors, latitude, leisure time physical activity), diet, and other characteristics (e.g., age, adiposity, self-reported race, smoking, alcohol drinking) have been shown to account for about 20–40% of the variability in circulating 25(OH)D [3], [7], [13], [24], [26], [36], [37]. To date, the estimated contribution of several identified SNPs to variation in 25(OH)D is generally less than 5% [11]–[13], although with further investigation this should increase. Among African Americans in our study, the genotype score based on three SNPs alone could account for 4.6% of the variation in 25(OH)D, whereas UVR score alone could account for 11.9% and diet 4.8%. It is important to note that the highest genotype score among African Americans was associated with an average reduction in serum 25(OH)D of 7.1 ng/mL; in context, this is not trivial given that mean serum 25(OH)D levels among African Americans are typically in the range of 10–20 ng/mL [7], [24]–[28].

We previously reported that, among African Americans, serum 25(OH)D levels decreased 1.0 ng/mL with each 10% increase in African ancestry [30]. We did not find evidence in this analysis, however, that the SNPs examined would account for these findings. Of the three SNPs that were associated with vitamin D levels, African Americans across the spectrum of ancestry had similar genotypes. This was contrary to our expectation that genotypes associated with the lowest 25(OH)D levels might be more common in individuals with the highest African ancestry. Our earlier findings may be a result of genetic variation not captured in this specific analysis, or skin color gradations along the spectrum of African ancestry, or both.

Our study is one of the first evaluations in African Americans of genetic determinants of vitamin D levels, and to our knowledge the first to evaluate how genotypes associated with vitamin D status vary in relation to African ancestry. Another strength of this investigation is its base in a generalizable population rather than patient groups. One limitation of the study is our reliance on a single measurement of 25(OH)D as representative of the subjects' general vitamin D status. However, in a separate analysis of 225 SCCS participants who provided two blood samples 1 to 3 years apart, we found a very high correlation (r = 0.91, p<0.001) between the two measured 25(OH)D levels (unpublished). The small size of our study and the lack of a replication group are additional limitations, and it will be important to seek replication of these findings in larger samples of African Americans.

In conclusion, we found that common genetic variation does play a role in determining 25(OH)D levels in African Americans, and we were able to replicate key recent findings regarding *GC* and *CYP27B1* SNPs reported from populations of European descent, which were paradoxically not evident in our Caucasian study group. Establishing a genetic underpinning of vitamin D levels among African Americans who are at particularly high risk for vitamin D deficiency would be an important advancement in our understanding of the drivers of vitamin D status and may provide further insights into the causes of certain racial health disparities. As the current body of work moves forward, it will be important to include discovery stage GWAS and larger candidate gene studies among African Americans. Such work may enable the identification of subgroups of African Americans especially in need of vitamin D-related interventions.

## Materials and Methods

### Ethics statement

SCCS participants provided written informed consent, and protocols were approved by Institutional Review Boards at Vanderbilt University and Meharry Medical College.

### Study population

The SCCS is a prospective cohort study of cancer risk disparities related to race, socioeconomic status, and other factors. Men and women aged 40–79 were enrolled across 12 southeastern US states between 2002 and 2009, largely by in-person recruitment at community health centers [38]. With approximately 86,000 participants enrolled, African Americans comprise two-thirds of the cohort. For the analyses herein, from SCCS participants who enrolled from March 2002–October 2004 and donated a baseline blood sample (N = 12,162), 792 were randomly selected using a 2×2×3×3 factorial design, with 22 individuals selected within each of the 36 strata defined by self-reported race (African American/non-Hispanic white), sex, smoking status (current former never), and body mass index (BMI, 18–24.99 kgm^2^, 25–29.99 kgm^2^, 30–45 kgm^2^). This design provided a balanced distribution across these factors which allowed for racial comparisons unconfounded by sex, smoking, and BMI, and avoided a population heavily enriched with current smokers and obese individuals (as obesity and current smoking is highly prevalent in the cohort).

### Baseline data and blood collection

Baseline data were collected using a computer-assisted, in-person interview conducted at the time of enrollment. The interview covered demographics, health history, anthropometrics, diet, and a wide range of potential cancer risk factors (questionnaire available at www.southerncommunitystudy.org). Dietary information was collected using a validated food frequency questionnaire developed specifically for the SCCS [39], [40]. Venous blood samples (20 mL) were collected during the baseline interview within the community health center, kept refrigerated and shipped cold overnight to Vanderbilt University where they were centrifuged the next day and stored at −80°C to await analysis.

### SNP Selection and Genotyping

For *VDR* genotyping (completed in 2008), the Caucasian (CEU) and African (YRI) populations in the International HapMap project were the primary data source for the selection of tagging SNPs [41]. These SNPs were selected to cover variants based on a linkage disequilibrium (LD) parameter of r^2^>0.8 in the *VDR* gene ±10 kb and a minor allele frequency (MAF) ≥0.05 for either the CEU or YRI populations. Functional SNPs were forced into the selection process. Three separate sets of SNPs were grouped as those tagging Caucasians only, those tagging Africans only, and those tagging both populations. All SNPs were scored for the ability to perform well on the Illumina GoldenGate genotyping platform using an Illumina in-house algorithm (Illumina Inc., San Diego, CA). The LDSelect algorithm was then run separately for the CEU and YRI data using an r^2^ cut-off of 0.8 [42]. When multiple tagging SNPs were found for an LD bin, those SNPs designated as tagging SNPs in both populations were preferentially selected for efficiency. Fifty-nine SNPs were thus selected for the *VDR* gene.

For *GC* (genotyped in 2010 along with *CYP2R1*, *CYP24A1*, and *CYP27B*) tagging SNPs were selected by analyzing the HapMap II+III genotype data (release 27) from CEU or YRI populations using the pairwise algorithm of Tagger function [43] implemented in HaploView version 4 [44]. These tagging SNPs covered variants based on an LD parameter of r^2^>0.8 in the *GC* gene ±5 kb and a MAF ≥0.05 for either CEU or YRI. Literature-reported or potentially functional SNPs in *GC* were forced into the SNP selection process. Thirty-seven SNPs were thus selected for the *GC* gene. We also selected 11 SNPs in *CYP2R1*, *CYP24A1*, and *CYP27B1* solely based on their reported associations with 25(OH)D in other populations (*CYP2R1*: rs10500804, rs1562902, rs10741657, rs10766197, rs2060793; *CYP24A1*: rs17219315, rs2244719, rs2296241; *CYP27B1*: rs10877012, rs4646536, rs703842) [11], [33]–[35], [45]–[47].

Genomic DNA was extracted from buffy coat using Qiagen's DNA purification kits (Qiagen, Valencia, CA) according to the manufacturer's instructions, and genotyping was carried out at Vanderbilt University using the Illumina GoldenGate genotyping platform (Illumina Inc., San Diego, CA) for the *VDR* gene and the Sequenom MassARRAY system (Sequenom, San Diego, CA) and TaqMan assays (Applied Biosystems, Carlsbad, CA) for the *GC*, *CYP2R1*, *CYP24A1*, and *CYP27B1* genes.

For *VDR*, 12 SNPs were excluded due to call rates <95%, leaving 47 SNPs for analysis. Blinded QC samples (N = 29) and another 171 pairs of duplicated samples were included in this genotyping run and the consistency rate was 99.9%. For *GC*, *CYP2R1*, *CYP24A1*, and *CYP27B1*, 47 of the 48 selected SNPs were designed in two Sequenom assays (29 and 18 SNPs, respectively) using the MassARRAY Assay Design 4.0 software, and 42 SNPs were successfully genotyped using the MassARRAY system (Sequenom, San Diego, CA) with call rates >97%. Genotype concordance of 36 pairs of duplicated samples was 100% and was 96.5%–100% (average 99.8%) for the 75 HapMap samples comparing genotyping data obtained from the current study with data obtained from the HapMap project. Genotypes of two SNPs (rs222054, rs1491719) which failed in the MassARRAY assay were imputed using IMPUTE2 [48], with the corresponding YRI or CEU HapMap phased haplotypes (release 22, build 36) as a reference panel. The imputation quality was very high for both SNPs, with probabilities for rs222054 and rs1491719 in African Americans being 95.4% and 86.9%, respectively, and in Caucasians 97.0% and 99.5%, respectively. Three additional SNPs that failed in MassARRAY assays (rs4588, rs2282679, rs155563) were genotyped using a TaqMan assay on a PRISM 7900HT Sequence Detector (Applied Biosystems, Carlsbad, CA). Call rates for these three SNPs ranged from 95.6% to 99.9%; the concordance rates were 100% for the 36 blinded duplicate samples and for the 75 HapMap samples comparing genotyping data obtained from the current study with data obtained from the HapMap project. One SNP in the *CYP27B1* gene (rs703842) failed in both Sequenom MassARRAY and Taqman assays and could not be imputed well for African Americans, and thus was dropped from the study.

### Ancestry estimation

A set of 276 ancestry informative SNPs compiled as described previously [30] allowed the estimation of African and European ancestry using a Bayesian clustering approach implemented using STRUCTURE software, version 2.2.3. [49], [50].

### Measurement of serum 25(OH)D and external sources of vitamin D

Blinded serum 25(OH)D measurements were performed at the Medical University of South Carolina using a radioimmunoassay method associated with high intra-assay reliability [51]. In our study samples, the coefficients of variation on duplicate, blinded quality control samples in two batches were 6.7% and 7.4%.

Dietary intake of vitamin D was estimated from the reported intake of the major food sources from the SCCS food frequency questionnaire: milk, cold cereal, tuna, eggs, multivitamins and calcium supplements. Individual measures of sun exposure were not available. However, as a surrogate we used ground ultraviolet radiation (UVR) measurements from the National Oceanic and Atmospheric Administration UV station geographically closest to the subject's residence (http:www.cpc.ncep.noaa.govproductsstratosphereuv_index). These UVR measurements are converted to an index that estimates the erythemal intensity, with scores ranging from 0 (none) to 11+ (extreme). We used the average of the UVR scores recorded at the monitoring station during the 3-month period preceding each participant's enrollment in the study, which we have previously shown to be highly correlated with SCCS participant serum 25(OH)D levels [24].

### Statistical Analysis

Of the 792 subjects selected for this study, 34 (4.3%) were excluded because either 25(OH)D could not be measured, genotyping could not be performed, or ancestry estimates were highly discordant with self-reported race. This left 758 subjects (379 African American, 379 Caucasian) for analysis.

Mean serum 25(OH)D values were computed for African American and Caucasian participants within each group of homozygous referent (HR), heterozygous (HET), and homozygous variant (HV) genotype for each SNP. HR was selected to be the most common race-specific homozygous genotype. The association between genotype and serum 25(OH)D level was first evaluated by linear regression analyses that modeled 25(OH)D as the dependent variable and genotype (i.e., with values of 0 for HR, 1 for HET, and 2 for HV) as the independent variable, with adjustment for sex and percentage African ancestry to account for potential population stratification [52]. In cases where less than 10 subjects were HV or HET combined, regression analysis was not performed. Szroeter's rank test for heteroskedasticity and normal probability plots of the residuals indicated that transformation of 25(OH)D in the regression models was not required.

For the 18 candidate SNPs identified from the literature (see footnotes to Table S1), a p-value cutoff of 0.05 was used to determine statistical significance for an association with 25(OH)D. For the tagging SNPs chosen for *GC* and *VDR*, in order to maintain a familywise α error level of 0.05, a Bonferroni correction was made based on 30 comparisons for *GC* and 47 for *VDR* (under the assumption that each gene represents a separate hypothesis [53]), resulting in p-value cutoffs of 1.66×10^−3^ and 1.06×10^−3^, respectively, denoting statistical significance. Testing for HWE for each SNP in African Americans and Caucasians was carried out using *PLINK* software [54].

SNPs found to be significantly associated with serum levels of 25(OH)D were examined in more detail as described below using a codominant inheritance model with indicator variables for each genotype unless fewer than 5 subjects were HV, in which case a dominant model was used that combined subjects with HV and HET.

We assessed whether genotypes associated with 25(OH)D levels varied in relation to African ancestry by calculating their frequency across strata defined by tertiles of African ancestry. We also assessed the predictive value of genetic factors on circulating 25(OH)D levels by comparing regression models with environmental predictors of 25(OH)D (UVR score, dietary intake of vitamin D, sex, age, African ancestry, body mass index, and smoking) with and without the SNP data. UVR score was favored as a covariate over season of blood collection, because the UVR score is informative for season as well as geographical location.

To evaluate the potential combined effects of SNPs identified as being significantly associated with serum 25(OH)D, a genotype score approach was used [12] where the score was equal to the sum of the number of risk alleles across the SNPs. The genotype score was evaluated in relation to serum 25(OH)D levels as well as to the risk of vitamin D insufficiency, which was defined as a serum 25(OH)D level below 20 ng/mL [9]. Odds ratios (OR) and 95% confidence intervals (CI) for vitamin D insufficiency were estimated using unconditional logistic regression models adjusted for sex and African ancestry.

## Supporting Information

Table S1 **Genotype frequency for SNPs in** ***GC*** **,** ***VDR*** **,** ***CYP2R1*** **,** ***CYP24A1*** **,** ***CYP27B1*** **, and their association with serum 25(OH)D among African American and Caucasian SCCS participants.**
(DOC)
