# Supplementary material for: Common Variation in Vitamin D Pathway Genes Predicts Circulating 25-Hydroxyvitamin D Levels among African Americans
Source: PLoS One. 2011 Dec 21;6(12):e28623. doi: 10.1371/journal.pone.0028623 (PMC3244405; doi:10.1371/journal.pone.0028623)
Supplement: Table S1 — Genotype frequency for SNPs in GC , VDR , CYP2R1 , CYP24A1 , CYP27B1 , and their association with serum 25(OH)D among African American and Caucasian SCCS participants. (DOC) [file pone.0028623.s001.doc]

**Table S1. Genotype frequency for SNPs in *GC*, *VDR*, *CYP2R1*, *CYP24A1*, *CYP27B1***, and their association with serum 25(OH)D among African American and Caucasian SCCS participants.

| **Gene** | **dbSNP ID** | **Chr** | **Position^a^** | **African Americans (N=379)** | | | | | **Caucasians (N=379)** | | | | |
| --- | --- | --- | --- | --- | --- | --- | --- | --- | --- | --- | --- | --- | --- |
|  |  |  |  | Homozygous referent (HR), Heterozygous (HET), Homozygous variant (HV)^a^ (*N in parentheses*) | Mean serum 25(OH)D, ng/mL | | | p-value^b^ | Homozygous referent (HR), Heterozygous (HET), Homozygous variant (HV)^a^ (*N in parentheses*) | Mean serum 25(OH)D, ng/mL | | | p-value^b^ |
|  |  |  |  |  | HR | HET | HV |  |  | HR | HET | HV |  |
| ***GC*** | rs12512631^c^ | 4 | 72820195 | TT (163), CT (166), CC (48) | 17.9 | 17.1 | 17.3 | 0.47 | TT (149), CT (180), CC (47) | 26.9 | 26.9 | 28.7 | 0.47 |
|  | rs222054^d^ | 4 | 72823163 | CC (229), CG (92), GG (6) | 16.7 | 18.2 | 22.4 | 0.17 |  |  |  |  |  |
|  | rs1352841 | 4 | 72824034 | TT (252), CT (113), CC (11) | 17.9 | 16.5 | 18.2 | 0.24 | TT (369), CT (7), CC (0) | 27.3 | 20.3 |  |  |
|  | rs17383291 | 4 | 72824274 | TT (366), GT(10), GG (0) | 17.5 | 13.9 |  | 0.15 | TT (329), GT(44), GG (3) | 27.1 | 28.3 | 21.5 | 0.87 |
|  | rs222049 | 4 | 72824819 | GG (285), CG (83), CC (9) | 17.4 | 17.5 | 21.3 | 0.25 | GG (322), CG (50), CC (4) | 27.4 | 25.8 | 23.0 | 0.26 |
|  | rs705117 | 4 | 72826979 | CC (230), CT (121), TT (25) | 17.3 | 17.8 | 16.7 | 0.55 | CC (12), CT (101), TT (263) | 27.5 | 26.5 | 27.5 | 0.64 |
|  | rs2282679^c^ | 4 | 72827247 | TT (317), GT (57), GG (1) | 17.7 | 15.5 | 9.4 | **0.03** | TT (199), GT (150), GG (26) | 27.5 | 27.0 | 26.9 | 0.66 |
|  | rs1491710 | 4 | 72827958 | AA (128), AC (160), CC (89) | 18.8 | 16.6 | 17.3 | 0.17 | AA (367), AC (9), CC (0) | 27.2 | 27.4 |  |  |
|  | rs842999^e^ | 4 | 72830554 | GG (292), CG (73), CC (6) | 17.1 | 18.6 | 18.1 | 0.50 | GG (67), CG (176), CC (96) | 27.1 | 26.6 | 28.8 | 0.35 |
|  | rs222043 | 4 | 72830563 | GG (293), AG (76), AA (7) | 17.3 | 18.2 | 19.0 | 0.32 | GG (320), AG (53), AA (3) | 27.5 | 25.4 | 25.2 | 0.25 |
|  | rs1491709 | 4 | 72832430 | GG (317), AG (59), AA (1) | 17.3 | 18.8 | 8.6 | 0.45 | GG (325), AG (48), AA (2) | 27.2 | 26.9 | 24.4 | 0.78 |
|  | rs9016 | 4 | 72837160 | CC (361), CT (16), TT (0) | 17.2 | 24.0 |  | 0.007 | CC (377), CT (0), TT (0) | 27.2 |  |  |  |
|  | rs4588^c^ | 4 | 72837187 | GG (311), GT (66), TT (1) | 17.6 | 16.6 | 9.4 | 0.21 | GG (200), GT (151), TT (28) | 27.5 | 27.0 | 26.2 | 0.55 |
|  | rs7041^c^ | 4 | 72837198 | AA (286), AC (83), CC (7) | 17.5 | 17.1 | 17.9 | 0.49 | AA (73), AC (184), CC (118) | 26.7 | 26.7 | 28.2 | 0.38 |
|  | rs17830803 | 4 | 72837849 | GG (319), GC (55), CC (1) | 17.3 | 18.6 | 22.2 | 0.25 | GG (320), GC (50), CC (3) | 27.3 | 25.9 | 25.2 | 0.42 |
|  | rs4752 | 4 | 72841430 | AA (208), AG (136), GG (33) | 17.8 | 17.0 | 17.6 | 0.79 | AA (374), AG (3), GG (0) | 27.1 | 42.0 |  |  |
|  | rs10488854 | 4 | 72842999 | CC (280), CT (84), TT (13) | 17.5 | 17.6 | 15.6 | 0.70 | CC (373), CT (4), TT (0) | 27.3 | 20.4 |  |  |
|  | rs1491719 | 4 | 72843365 | TT (126), CT (96), CC (29) | 18.3 | 17.2 | 18.6 | 0.92 | TT (324), CT (51), CC (3) | 27.5 | 25.6 | 25.2 | 0.29 |
|  | rs188812 | 4 | 72846548 | AA (247), AT (115), TT (14) | 17.4 | 17.5 | 18.4 | 0.93 | AA (313), AT (59), TT (3) | 26.9 | 27.8 | 38.4 | 0.19 |
|  | rs6835052 | 4 | 72848292 | AA (333), AG (43), GG (1) | 17.3 | 18.7 | 9.4 | 0.45 | AA (376), AG (0), GG (0) | 27.2 |  |  |  |
|  | rs3775152 | 4 | 72849506 | CC (172), AC (151), AA (54) | 17.0 | 18.4 | 16.6 | 0.44 | CC (370), AC (6), AA (0) | 27.1 | 28.0 |  |  |
|  | rs3737549 | 4 | 72850532 | GG (255), AG (105), AA (17) | 17.2 | 18.2 | 16.7 | 0.33 | GG (372), AG (5), AA (0) | 27.3 | 21.6 |  |  |
|  | rs705124 | 4 | 72851507 | GG (208), AG (125), AA (44) | 17.6 | 16.8 | 18.5 | 0.89 | GG (315), AG (57), AA (4) | 26.8 | 29.1 | 34.3 | 0.06 |
|  | rs222014 | 4 | 72851795 | CC (312), CT (60), TT (5) | 17.4 | 17.4 | 25.8 | 0.48 | CC (315), CT (58), TT (3) | 26.9 | 27.9 | 38.4 | 0.18 |
|  | rs222017 | 4 | 72854376 | TT (289), AT (82), AA (6) | 17.2 | 17.8 | 24.8 | 0.32 | TT (315), AT (59), AA (3) | 26.9 | 28.0 | 38.4 | 0.17 |
|  | rs222020^c^ | 4 | 72855136 | CC (127), CT (171), TT (78) | 18.3 | 17.3 | 16.4 | 0.09 | CC (7), CT (94), TT (275) | 29.7 | 27.0 | 27.2 | 0.86 |
|  | rs222023 | 4 | 72855944 | GG (116), AG (165), AA (96) | 17.1 | 17.0 | 18.7 | 0.13 | GG (336), AG (39), AA (2) | 27.5 | 24.9 | 24.4 | 0.17 |
|  | rs16847015 | 4 | 72857688 | CC (269), AC (92), AA (14) | 17.1 | 18.6 | 17.9 | 0.16 | CC (340), AC (35), AA (2) | 27.3 | 26.4 | 24.4 | 0.55 |
|  | rs16847019 | 4 | 72857866 | GG (289), AG (75), AA (11) | 17.8 | 16.8 | 13.2 | 0.16 | GG (374), AG (1), AA (0) | 27.2 | 16.6 |  |  |
|  | rs1491718 | 4 | 72860143 | TT (320), CT (56), CC (3) | 17.3 | 17.5 | 31.1 | 0.31 | TT (317), CT (59), CC (3) | 26.9 | 28.0 | 38.4 | 0.17 |
|  | rs1155563^c^ | 4 | 72862352 | TT (299), CT (58), CC (2) | 17.4 | 17.2 | 11.7 | 0.47 | TT (186), CT (151), CC (29) | 27.0 | 27.5 | 26.4 | 0.88 |
|  | rs2298849^c^ | 4 | 72867715 | AA (139), AG (172), GG (61) | 16.4 | 17.5 | 20.4 | **0.008** | AA (240), AG (120), GG (10) | 27.1 | 27.0 | 27.3 | 0.97 |
|  | rs3733359 | 4 | 72868638 | GG (230), AG (119), AA (28) | 17.1 | 16.9 | 22.5 | 0.05 | GG (329), AG (47), AA (1) | 27.4 | 25.5 | 32.6 | 0.42 |
|  | rs16847024 | 4 | 72869543 | CC (321), CT (51), TT (4) | 17.2 | 19.5 | 18.2 | 0.14 | CC (372), CT (3), TT (0) | 27.3 | 16.8 |  |  |
|  | rs16847028 | 4 | 72870389 | GG (270), AG (99), AA (7) | 17.0 | 18.4 | 22.1 | 0.09 | GG (294), AG (78), AA (4) | 27.1 | 27.3 | 36.7 | 0.44 |
|  | rs1873590 | 4 | 72872344 | AA (278), AG (90), GG (9) | 17.3 | 18.5 | 13.1 | 0.80 | AA (373), AG (3), GG (0) | 27.0 | 42.7 |  |  |
|  | rs6817912 | 4 | 72872569 | CC (312), CT (58), TT (7) | 17.3 | 18.4 | 19.4 | 0.33 | CC (328), CT (47), TT (2) | 27.4 | 25.7 | 22.8 | 0.28 |
|  |  |  |  |  |  |  |  |  |  |  |  |  |  |
| ***VDR*** | rs11608702 | 12 | 46515035 | AA (229), AT (127), TT (23) | 16.8 | 18.7 | 17.2 | 0.15 | AA (153), AT (172), TT (54) | 26.2 | 28.3 | 26.5 | 0.33 |
|  | rs11834903 | 12 | 46516003 | GG (330), CG (48), CC (1) | 17.5 | 17.2 | 10.9 | 0.93 | GG (378), CG (1), CC (0) | 27.2 | 35.8 | - |  |
|  | rs12721364 | 12 | 46517697 | GG (365), AG (14), AA (0) | 17.6 | 13.5 | - | 0.16 | GG (267), AG (107), AA (4) | 27.3 | 27.2 | 20.3 | 0.74 |
|  | rs7965281 | 12 | 46517877 | AA (145), AG (171), GG (63) | 17.9 | 17.1 | 17.4 | 0.59 | AA (90), AG (197), GG (92) | 26.1 | 28.5 | 25.3 | 0.64 |
|  | rs2525045 | 12 | 46519287 | GG (278), AG (97), AA (4) | 17.6 | 17.4 | 12.3 | 0.65 | GG (378), AG (1), AA (0) | 27.2 | 24.2 | - |  |
|  | rs881383 | 12 | 46519754 | GG (364), AG (14), AA (1) | 17.4 | 18.4 | 24.3 | 0.43 | GG (379), AG (0), AA (0) | 27.2 | - | - |  |
|  | rs11574143 | 12 | 46521184 | GG (318), AG (58), AA (3) | 17.5 | 17.2 | 15.1 | 0.42 | GG (313), AG (66), AA (0) | 27.1 | 27.6 | - | 0.84 |
|  | rs11574141 | 12 | 46521297 | GG (341), CG (37), CC (1) | 17.4 | 18.5 | 0.5 | 0.56 | GG (378), CG (1), CC (0) | 27.2 | 35.8 | - |  |
|  | rs11574138 | 12 | 46521969 | AA (306), AG (71), GG (2) | 17.5 | 17.2 | 17.8 | 0.77 | AA (379), AG (0), GG (0) | 27.2 | - | - |  |
|  | rs11574110 | 12 | 46526031 | GG (369), AG (9), AA (0) | 17.5 | 15.4 | - |  | GG (379), AG (0), AA (0) | 27.2 | - | - |  |
|  | rs1544410 | 12 | 46526102 | GG (205), AG (145), AA (29) | 17.2 | 17.8 | 17.6 | 0.43 | GG (130), AG (190), AA (59) | 27.2 | 27.7 | 25.5 | 0.55 |
|  | rs12314197^d^ | 12 | 46528989 | AA (214), AG (141), GG (23) | 17.1 | 18.1 | 16.3 | 0.62 |  |  |  |  |  |
|  | rs7962898 | 12 | 46529104 | GG (173), AG (167), AA (39) | 17.5 | 17.0 | 18.9 | 0.83 | GG (84), AG (200), AA (95) | 26.5 | 28.1 | 25.7 | 0.58 |
|  | rs7967152 | 12 | 46530451 | CC (139), AC (177), AA (63) | 17.4 | 17.7 | 16.9 | 0.76 | CC (96), AC (201), AA (82) | 25.7 | 28.2 | 26.5 | 0.58 |
|  | rs2239185 | 12 | 46530826 | AA (122), AG (179), GG (76) | 17.3 | 17.5 | 17.4 | 0.94 | AA (95), AG (200), GG (83) | 25.7 | 28.2 | 26.4 | 0.60 |
|  | rs7975128 | 12 | 46532095 | GG (216), AG (141), AA (22) | 17.4 | 17.5 | 18.4 | 0.53 | GG (128), AG (194), AA (57) | 27.1 | 27.7 | 25.6 | 0.64 |
|  | rs11168264 | 12 | 46533130 | AA (205), AG (154), GG (20) | 17.5 | 17.6 | 16.0 | 0.99 | AA (377), AG (2), GG (0) | 27.2 | 30.0 | - |  |
|  | rs7305032 | 12 | 46536127 | AA (199), AG (148), GG (32) | 17.6 | 17.4 | 16.5 | 0.41 | AA (114), AG (191), GG (74) | 25.1 | 28.4 | 27.2 | 0.13 |
|  | rs11168266 | 12 | 46537800 | AA (119), AG (198), GG (61) | 17.6 | 17.5 | 17.0 | 0.49 | AA (110), AG (193), GG (75) | 25.1 | 28.3 | 27.1 | 0.15 |
|  | rs11168268 | 12 | 46538079 | AA (142), AG (167), GG (70) | 17.5 | 18.0 | 16.0 | 0.25 | AA (111), AG (194), GG (74) | 25.3 | 28.4 | 27.0 | 0.19 |
|  | rs12308082 | 12 | 46538406 | GG (330), AG (47), AA (2) | 17.2 | 19.1 | 12.7 | 0.26 | GG (378), AG (1), AA (0) | 27.2 | 35.8 | - |  |
|  | rs2853560 | 12 | 46538815 | GG (359), AG (20), AA (0) | 17.6 | 14.1 | - | 0.11 | GG (379), AG (0), AA (0) | 27.2 | - | - |  |
|  | rs2248098 | 12 | 46539623 | AA (111), AG (190), GG (78) | 17.1 | 17.8 | 17.2 | 0.84 | AA (88), AG (201), GG (90) | 26.6 | 28.1 | 25.6 | 0.55 |
|  | rs2239182 | 12 | 46541678 | GG (103), AG (190), AA (85) | 17.6 | 17.9 | 16.4 | 0.36 | GG (96), AG (199), AA (84) | 24.0 | 28.7 | 27.3 | 0.05 |
|  | rs2107301 | 12 | 46541837 | GG (233), AG (133), AA (13) | 17.6 | 17.3 | 16.3 | 0.50 | GG (197), AG (158), AA (24) | 26.8 | 28.3 | 23.2 | 0.82 |
|  | rs2239179 | 12 | 46544033 | AA (200), AG (142), GG (35) | 17.4 | 17.3 | 18.5 | 0.54 | AA (113), AG (200), GG (66) | 26.8 | 28.5 | 24.0 | 0.31 |
|  | rs11574065 | 12 | 46545292 | GG (362), AG (12), AA (1) | 17.4 | 17.3 | 22.2 | 0.68 | GG (377), AG (0), AA (0) | 27.2 | - | - |  |
|  | rs12717991 | 12 | 46545393 | GG (153), AG (173), AA (51) | 17.3 | 18.0 | 16.1 | 0.65 | GG (147), AG (181), AA (50) | 26.7 | 27.7 | 26.8 | 0.85 |
|  | rs12721370 | 12 | 46548340 | CC (329), AC (48), AA (2) | 17.5 | 17.5 | 16.7 | 0.67 | CC (303), AC (75), AA (0) | 27.3 | 26.8 | - | 0.68 |
|  | rs886441 | 12 | 46549231 | AA (136), AG (180), GG (62) | 17.0 | 17.7 | 17.8 | 0.60 | AA (243), AG (129), GG (7) | 26.8 | 28.0 | 25.7 | 0.44 |
|  | rs2189480 | 12 | 46550095 | CC (155), AC (165), AA (59) | 17.8 | 17.3 | 16.8 | 0.26 | CC (146), AC (178), AA (55) | 26.8 | 27.1 | 28.5 | 0.45 |
|  | rs3782905 | 12 | 46552434 | GG (245), CG (119), CC (15) | 17.4 | 17.4 | 19.2 | 0.37 | GG (169), CG (170), CC (40) | 27.8 | 27.3 | 24.1 | 0.13 |
|  | rs2239186 | 12 | 46555677 | AA (348), AG (28), GG (2) | 17.7 | 14.5 | 9.8 | 0.02 | AA (237), AG (129), GG (13) | 26.7 | 28.3 | 25.7 | 0.42 |
|  | rs2238136 | 12 | 46563980 | GG (309), AG (68), AA (2) | 17.2 | 18.8 | 17.2 | 0.32 | GG (202), AG (157), AA (19) | 26.9 | 27.3 | 30.2 | 0.43 |
|  | rs2853564 | 12 | 46564754 | AA (298), AG (76), GG (5) | 17.5 | 17.0 | 22.7 | 0.78 | AA (130), AG (196), GG (53) | 28.0 | 27.0 | 25.9 | 0.26 |
|  | rs4760648 | 12 | 46566932 | AA (113), AG (195), GG (67) | 18.2 | 17.2 | 17.1 | 0.63 | AA (67), AG (185), GG (124) | 27.7 | 27.1 | 27.0 | 0.78 |
|  | rs11168287 | 12 | 46571681 | AA (193), AG (154), GG (32) | 17.8 | 16.9 | 17.7 | 0.39 | AA (85), AG (200), GG (94) | 26.8 | 26.9 | 28.2 | 0.38 |
|  | rs4328262 | 12 | 46571915 | AA (166), AC (167), CC (42) | 18.0 | 17.2 | 17.2 | 0.65 | AA (124), AC (197), CC (58) | 26.5 | 27.2 | 28.7 | 0.22 |
|  | rs4334089 | 12 | 46572282 | AA (152), AG (170), GG (57) | 17.2 | 17.1 | 19.3 | 0.23 | AA (26), AG (155), GG (198) | 26.2 | 27.4 | 27.1 | 0.94 |
|  | rs11574027 | 12 | 46573640 | CC (369), AC (10), AA (0) | 17.4 | 19.6 |  | 0.66 | CC (372), AC (7), AA (0) | 27.2 | 27.8 |  |  |
|  | rs11168288 | 12 | 46574053 | GG (279), AG (94), AA (6) | 17.4 | 17.6 | 17.5 | 0.57 | GG (378), AG (1), AA (0) | 27.2 | 34.1 |  |  |
|  | rs10875695 | 12 | 46579304 | AA (94), AC (193), CC (92) | 17.5 | 16.9 | 18.5 | 0.47 | AA (26), AC (156), CC (197) | 27.0 | 27.2 | 27.2 | 0.98 |
|  | rs11168292 | 12 | 46579872 | GG (285), CG (90), CC (4) | 17.5 | 17.5 | 15.8 | 0.74 | GG (171), CG (171), CC (37) | 27.9 | 26.7 | 26.3 | 0.24 |
|  | rs7299460 | 12 | 46582535 | AA (231), AG (133), GG (15) | 17.6 | 17.1 | 18.7 | 0.56 | AA (32), AG (167), GG (180) | 27.6 | 27.0 | 27.3 | 0.95 |
|  | rs4516035^c^ | 12 | 46586093 | AA (322), AG (53), GG (4) | 17.4 | 17.7 | 19.4 | 0.91 | AA (125), AG (191), GG (62) | 27.4 | 27.1 | 26.9 | 0.66 |
|  | rs11568820 | 12 | 46588812 | AA (243), AG (122), GG (14) | 17.4 | 17.2 | 19.6 | 0.74 | AA (15), AG (129), GG (235) | 25.0 | 26.7 | 27.6 | 0.33 |
|  | rs4077869 | 12 | 46591911 | AA (126), AG (169), GG (84) | 18.0 | 16.9 | 17.7 | 0.93 | AA (377), AG (2), GG (0) | 27.2 | 24.0 |  |  |
|  |  |  |  |  |  |  |  |  |  |  |  |  |  |
| ***CYP2R1*** | rs10500804^c^ | 11 | 14866849 | TT (275), GT (95), GG (6) | 17.4 | 18.0 | 12.5 | 0.83 | TT (131), GT (178), GG (67) | 27.4 | 27.6 | 25.7 | 0.37 |
|  | rs10741657^c^ | 11 | 14871454 | GG (215), AG (135), AA (26) | 16.8 | 18.3 | 18.0 | 0.19 | GG (135), AG (186), AA (56) | 27.0 | 26.5 | 29.8 | 0.24 |
|  | rs2060793^c^ | 11 | 14871886 | GG (164), AG (169), AA (44) | 16.5 | 18.4 | 17.6 | 0.17 | GG (135), AG (186), AA (56) | 27.0 | 26.5 | 29.8 | 0.24 |
|  | rs1562902^c^ | 11 | 14874792 | TT (104), CT (181), CC (92) | 16.4 | 17.9 | 17.8 | 0.26 | TT (110), CT (199), CC (67) | 26.3 | 27.7 | 27.3 | 0.48 |
|  | rs10766197^c^ | 11 | 14878456 | GG (272), AG (95), AA (7) | 17.4 | 17.8 | 15.8 | 0.84 | GG (112), AG (184), AA (81) | 26.7 | 27.9 | 26.2 | 0.89 |
|  |  |  |  |  |  |  |  |  |  |  |  |  |  |
| ***CYP24A1*** | rs2244719^c^ | 20 | 52216265 | TT (204), CT (146), CC (26) | 17.5 | 17.2 | 18.8 | 0.79 | TT (100), CT (191), CC (82) | 28.2 | 27.5 | 25.0 | 0.06 |
|  | rs2296241^c^ | 20 | 52219626 | AA (109), AG (182), GG (86) | 17.5 | 17.7 | 17.0 | 0.40 | AA (110), AG (177), GG (90) | 28.5 | 26.0 | 27.9 | 0.63 |
|  | rs17219315^c^ | 20 | 52221853 | AA (371), AG (5), GG (0) | 17.3 | 25.7 |  |  | AA (361), AG (15), GG (0) | 27.2 | 26.2 |  | 0.66 |
|  |  |  |  |  |  |  |  |  |  |  |  |  |  |
| ***CYP27B1*** | rs4646536^c^ | 12 | 56444255 | AA (205), AG (147), GG (25) | 17.0 | 18.0 | 18.2 | 0.24 | AA (177), AG (169), GG (28) | 27.0 | 27.1 | 27.7 | 0.80 |
|  | rs10877012^c^ | 12 | 56448352 | GG (291), GT (82), TT (3) | 16.9 | 18.9 | 26.5 | **0.02** | GG (188), GT (161), TT (27) | 27.3 | 27.0 | 28.0 | 0.99 |

^a^ Base position on chromosome (Chr) and allele naming of each single nucleotide polymorphism (SNP) are based on forward strand according to human genome assembly 18 (March 2006, NCBI Build 36.1)

^b^ p-value for the association between the SNP and 25(OH)D levels from race-stratified linear regression models adjusted for sex and level of African ancestry.

^c^ SNPs identified from the literature as being significantly associated with 25(OH)D levels.

^d^ SNP deviated from HWE in Caucasians

^e^ rs842999 has three alleles (ACG). For the purposes of this analysis, subjects with the A allele were excluded, as the A allele was very rare (only 5 African American and 37 white participants had genotypes involving the A allele).
